# Co-Factor Binding Confers Substrate Specificity to Xylose Reductase from *Debaryomyces hansenii*

**DOI:** 10.1371/journal.pone.0045525

**Published:** 2012-09-26

**Authors:** Dipanwita Biswas, Vaibhav Pandya, Appu Kumar Singh, Alok K. Mondal, S. Kumaran

**Affiliations:** Council of Scientific and Industrial Research (CSIR), Institute of Microbial Technology, Chandigarh, India; Aligarh Muslim University, India

## Abstract

Binding of substrates into the active site, often through complementarity of shapes and charges, is central to the specificity of an enzyme. In many cases, substrate binding induces conformational changes in the active site, promoting specific interactions between them. In contrast, non-substrates either fail to bind or do not induce the requisite conformational changes upon binding and thus no catalysis occurs. In principle, both lock and key and induced-fit binding can provide specific interactions between the substrate and the enzyme. In this study, we present an interesting case where cofactor binding pre-tunes the active site geometry to recognize only the cognate substrates. We illustrate this principle by studying the substrate binding and kinetic properties of Xylose Reductase from *Debaryomyces hansenii* (*Dh*XR), an AKR family enzyme which catalyzes the reduction of carbonyl substrates using NADPH as co-factor. *Dh*XR reduces D-xylose with increased specificity and shows no activity towards “non-substrate” sugars like L-rhamnose. Interestingly, apo-*Dh*XR binds to D-xylose and L-rhamnose with similar affinity (K_d_∼5.0–10.0 mM). Crystal structure of apo-*Dh*XR-rhamnose complex shows that L-rhamnose is bound to the active site cavity. L-rhamnose does not bind to holo-*Dh*XR complex and thus, it cannot competitively inhibit D-xylose binding and catalysis even at 4–5 fold molar excess. Comparison of K_d_ values with K_m_ values reveals that increased specificity for D-xylose is achieved at the cost of moderately reduced affinity. The present work reveals a latent regulatory role for cofactor binding which was previously unknown and suggests that cofactor induced conformational changes may increase the complimentarity between D-xylose and active site similar to specificity achieved through induced-fit mechanism.

## Introduction

Non-covalent binding of substrate to the active site of the enzyme is the first step in enzyme catalyzed reactions [1]–[3]. The correct fitting of incoming substrate into active site pocket is by steered mainly by charge and shape complimentarity between substrate and active site of the enzyme [1], [4]–[6]. The formation of enzyme-substrate complex is best described by induced fit model which suggests that binding of the substrate induces specific conformational changes within the active site and the productive enzyme-substrate complex formation is determined by these conformational dynamics [7]–[10]. A structurally related “non-substrate” molecule can also bind the active site, but binding may not result in the formation of catalytically productive enzyme-substrate complex due to different conformational dynamics induced by the “non-substrate” molecule [8], [11], [12]. Structurally related “non-substrate” molecules pose great challenges when enzyme has to selectively catalyze the substrate from a mixture of compounds, a condition often encountered under physiological conditions [11]. To achieve specificity towards its substrate, enzymes have evolved with a number of mechanisms to discriminate between substrate and competing non-substrate molecules [11]–[13]. A recent study noted that conformational deformation of the active site upon substrate binding may serve as one of the proof reading mechanism [13]. Specificity may also be achieved if enzyme exists in ensemble of conformations and substrate selectively binds to one of them and shifts equilibrium towards that conformation [14]–[17]. Another mechanism by which enzymes catalyze their cognate substrates specifically is by pre-tuning their active site structure for selectively binding the correct substrate, the area which is still underexplored [11], [18]–[20].

Pre-tuning of active site/functional sites in proteins by allosteric interactions between the active site and allosteric ligand binding site have been reported for enzymes involved in DNA metabolism and signaling [18], [19]. Allosteric ligands which bind to different sites on the enzyme alter the active site conformation and hence, alter enzyme activity [20]. Many cofactors act as allosteric ligands and in particular, mono- and di- nucleotide based cofactors (ATP, GTP, NAD(P)H) control activities of enzymes allosterically [19,21]. It has been reported that binding of cofactors like NAD(P)H changes the structure of active site and cofactor binding is known to precede the substrate binding [22], [23]. In these cases, cofactors are considered as co-substrates because they share the active site with substrates and participate in catalysis by carrying out chemical reactions which cannot be performed by natural amino acids [24], [25]. Conformational changes induced by cofactors are assumed to facilitate proton transfer, electron delocalization during hydride ion transfer, and/or facilitate the release of products and oxidized cofactors [26]. Cofactors are also known to stabilize native conformation, assist folding, and play a role in oligomerization of proteins [22], [23], [27], [28]. Although it is intuitive that restructuring of active site may affect substrate binding, the impact of cofactor mediated structural changes on the substrate specificity in xylose reductases has not been studied. The rapid binding of cofactor and subsequent conformational changes preceding the substrate binding might suggest as if the active site was prepared for substrate binding [29]. Therefore, we speculated that the active site structure is pre-tuned by cofactor binding and active site selects substrate molecules in accordance with cofactor induced structural remodeling.

We tested this idea of cofactor binding mediated substrate selectivity by systematically examining the substrate recognition properties of apo- and holoenzyme of xylose reductase (*Dh*XR) from *Debaryomyces hansenii*. Cofactor associated conformational changes for xylose reductase (XR) is well characterized by structural approaches and details on kinetic properties of XR are available [29]–[32]. D-xylose is the second most abundant sugar present in the lignocellulosic biomass and fuel ethanol production from lignocellulosic biomass would be low cost alternative fuel [33]–[36]. *D. hansenii* can utilize both xylose and D-arabinose, and it is halotolerant as well as osmotolerant, and therefore it offers great promise in bio-ethanol production [37], [38]. Therefore detailed biochemical characterization on the substrate selectivity of this enzyme will aid in engineering efficient xylose utilizing XR for ethanol production. Using *Dh*XR as a model system, we tested the possible connection between cofactor associated active site restructuring and substrate selectivity.

Equilibrium binding studies of *Dh*XR with substrate, cofactor, and sugar have been examined and compared with kinetic activities of the enzyme. We have observed that dimeric apo-*Dh*XR binds a variety of sugars almost with similar affinity, but cofactor bound enzyme does not hydrolyze non-xylose substrates like L-rhamnose. Crystallography studies show that L-rhamnose binds to active site of apo-*Dh*XR. Site-directed mutagenesis of residues lining the active site cavity (D42, Y47, K76, H109, and N305) show that these residues are important for holo-*Dh*XR activity, but mutations do not reduce the binding affinity of apo-*Dh*XR significantly. Comparisons of parameters obtained from equilibrium and kinetic studies suggest that cofactor binding decreases the affinity for non-substrate sugars. This observation is also supported by results of kinetics performed in the presence of non-xylose sugars. In summary, cofactor binding provides additional screening mechanism for recognizing the substrate more specifically.

## Materials and Methods

### Materials

All chemicals and reagents were of analytical reagent grade and were procured from different commercial sources. D-xylose, D-ribose, D-arabinose, D-galactose, L-rhamnose, sucrose, xylitol, and Nicotinamide adenine dinucleotide phosphate (NADPH) are obtained from Sigma chemicals (USA).

### Cloning of DhXR and Generation of Mutants of *Dh*XR

The *Dh*XR ORF was PCR amplified from genomic DNA of *D. hansenii* CBS767 using pETXRf (NdeI site introduced) and pETXRr (XhoI site introduced). PCR was carried out using vent polymerase (NEB) and amplified product was cloned into NdeI and XhoI site of the expression vector pET28c. The resulting plasmid was sequenced and designated as p*Dh*XR. Five mutants of *Dh*XR (D42A, Y47A, K76A, H109A, and N305A) were generated by overlap extension PCR method using p*Dh*XR as a template and cloned into pET28c vector at NheI and XhoI site. A complete list of primers used for site directed mutagenesis is mentioned in Text S1. The positive clones were sequenced and transformed in BL21(DE3) expression strain.

### Protein Expression and Purification

For the expression of pET28c-*Dh*XR and mutant constructs, BL21(DE3) was used as an expression host. Protein expression was induced by 0.2 mM IPTG and induction was carried out at 25°C for 16 hrs at 180 rpm. Cultures were harvested and lysed by sonication for 30 minutes and the soluble fraction containing the desired protein was recovered by centrifugation. The N-terminally His-tagged *Dh*XR was purified using Ni-NTA affinity chromatography followed by gel-filtration chromatography on Hiprep 16/60 Sephacryl S-200 column (GE Healthcare). The purified protein was dialyzed against 50 mM potassium phosphate, pH 7.5, and 100 mM NaCl. The purified *Dh*XR was monitored in 10% SDS-PAGE gel followed by Coomassie brilliant blue R-250 staining. The purity of protein was found to be around 90–95%.

### Enzymatic Assay of XR with Different Sugars

The xylose reductase activities of recombinant *Dh*XR was determined spectrophotometrically by monitoring the change in A_340_ upon reduction of NADPH to NADP. The standard assay mixture contained 50 mM KPO_4_, pH 7.0, 100 mM sugar substrate, 0.3 mM NADPH, 0.18 µM of enzyme. Single point activity studies were performed at 40°C and all reactions were started by the addition of enzyme to a final volume of 0.8 ml (in standard assay condition). Different sugars used for the activity are, D-xylose, D-arabinose, D-ribose, D-galactose, L-rhamnose, sucrose and xylitol. Errors for the activity assays were calculated from triplicate experiments. The kinetic parameters were determined using a range of substrate concentrations. Here, the reaction was performed at 25°C and assay mixture contained 50 mM KPO_4_, pH 7.0, 0.15 mM NADPH, 0.18 µM of enzyme and varying substrate concentrations. Steady state kinetics data were fit to Michaelis-Menten model, v = V_max_ * [S]/([S]+K_m_), where V_max_, maximal velocity; K_m_, Michaelis-Menten constant; S, substrate concentration, and v, initial velocity.

### Fluorescence Titration Measurements

Titrations of *Dh*XR with ligands were examined by monitoring the intrinsic tryptophan fluorescence of *Dh*XR using a Varian spectrofluorometer. Experiments were performed in indicated buffers as mentioned in the text. The excitation wavelength and emission wavelengths for monitoring *Dh*XR-ligand interaction were 292 nm and 345 nm respectively. Slit widths were set to 5 nm for all experiments and PMT voltage was adjusted to get maximum signal for a given protein concentration. All experiments were done at 25.0±1°C. Initial readings of both the protein, *F_protein,0_* and buffer, *F_buff,0_* were taken, with *F_0_* = *F_protein,0_–F_buff,0_* defined as the initial fluorescence of the sample. The sample cuvette was then titrated with aliquots of ligands and mixed, and equilibrated for 3–4 minutes before measurement. Data points from five such measurements were averaged to obtain *F_ave,i_*. The relative fluorescence quenching upon sugar binding is defined as *Q_obs,i_* = *(F_0_–F_ave,i_)/F_0_*. All measurements were corrected for dilution, and inner filter effects.

### Analysis of Fluorescence Titrations for the Binding of Ligands to *Dh*XR

Binding of ligands to *Dh*XR was analyzed using two site binding model.
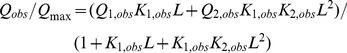
(1)where *Q_1_* and *Q_2_* are the fluorescence quenching corresponding to one and two ligands bound, respectively; L is concentration of free ligand in solution; *K_1,obs_* and *K_2,obs_* are association constants for the binding of the first and the second ligand molecule. Errors were calculated from fitting analysis of two independent experiments.

### X-ray Data Collection and Structure Determination

Purified *Dh*XR was crystallized at 18°C by sitting drop vapor diffusion method using ammonium sulphate screening suite (NeXtal Classics Suite-96, Qiagen Sciences, Maryland USA). 1.0 µL of protein (20 mg/mL) was mixed with 1.0 µL of reservoir solution and equilibrated against 80 µL of precipitant solution. Although crystals appeared in several conditions, good quality crystals were grown in 2.0 M ammonium sulfate, pH 7.5, 4 mM MgCl_2_ by mixing 2.0 µL protein solution containing 15.0 mg/mL with 2.0 µL of buffer. Apoenzyme crystals were then soaked in the mother liquor containing 50.0 mM L-rhamnose for 3–5 hours. Soaked crystals were then equilibrated in native solution containing 20% glycerol and flash cooled in liquid nitrogen. Diffraction data were collected at 100 K on the in-house MAR345 image plate detector mounted on a Rigaku MicroMax-007HF microfocus rotating anode X-ray generator. Diffraction data collected was processed, integrated, and scaled using HKL2000 suite [39]. Structures of apo-*Dh*XR and *Dh*XR in complex with L-rhamnose were solved by molecular replacement using the program PHASER and CCP4 suite [40], [41]. The apoform crystal structure of *Ct*XR (xylose reductase from *Candida tenuis*) (PDB code 1MI3), a homologue of *Dh*XR sharing 69% sequence identity was used as template for finding initial solution. Diffraction data between 20–5 Å were used to obtain initial solution. 5% of data were flagged for R_free_ and electron density maps were obtained after rigid body refinement. Initial models were subsequently refined using Phenix and many rounds of manual fitting and model refinement were done using COOT [42], [43].

## Results

### Characterization of *Dh*XR and its Interaction with Substrate, Product, and Cofactor

We examined the purified *Dh*XR using size exclusion chromatography and *Dh*XR eluted as a single peak with peak volume at 52 mL. Calibration for molecular mass determination was done by using protein standards (GE Healthcare): ferritin (440 kDa), catalase (232 kDa), aldolase (158 kDa), albumin (67 kDa) and ovalbumin (43 kDa). The molecular mass of *Dh*XR was estimated to be ∼71 kDa (Fig. S1) which is consistent with the molecular weight of a homo-dimer. Analyses of *Dh*XR sequence shows that known dimerization motifs, “SGAL”, “RLIEF”, “NPWDWK”, are found in the sequence of *Dh*XR [44]. Circular dichroism (CD) spectrum of *Dh*XR showed that it is folded and exhibits native like structure as expected from structural features for α/β proteins. (data not shown).

We examined the binding of D-xylose, xylitol, and NADPH to apo-*Dh*XR. Quenching of tryptophan fluorescence upon ligand binding was used as the signal for monitoring the extent of binding. Apo-*Dh*XR was excited at 292 nm and emission of tryptophan fluorescence was scanned between 300–375 nm (Fig. S2). Since NADPH shows significant absorption at 345 nm, NADPH binding was monitored at 375 nm in order to avoid any interference from cofactor absorption. Binding data can be described better by two non-identical binding sites model (eq 1, method) and binding isotherms are shown (Fig. S3 A–C). Intrinsic site-specific binding constants are estimated from the macroscopic binding constants obtained from fitting by removing the statistical factors as in eq (2).
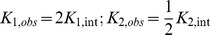
(2)


Two D-xylose units bind to apo-*Dh*XR dimer with different affinity. Affinity of first D-xylose, K_1,int_ = 1.9±0.6×10^2^ M^−1^, (K_d_ ∼5.3 mM) which is ∼4 times larger than the affinity for the second D-xylose molecule; K_2,int_ = 5.8±0.1×10^1^ M^−1^, (K_d_ = 17 mM). The Hill coefficient, n_H,_ estimated from the logarithmic plot is also less than 1 (∼0.68) indicating that the second monomer binds with reduced affinity (Fig. S4). Compared with substrate affinity, NADPH has much higher affinity for apo-*Dh*XR (K_1,int_ = 8.9±0.4×10^5^ M^−1^; K_d_∼1.0 µM) and the two intrinsic binding constants differ by a factor of ∼4 (table 1). In the absence of any information on ligand binding properties of apo-*Dh*XR, our results show that both substrate and product can bind in a cofactor independent manner.

**Table 1:** Determination of equilibrium binding constants for carbonyl substrates binding to wild type xylose reductase.

Substrate	Kd_1,int_ (mM)	Kd_2,int_ (mM)
D-xylose	5.3±2	17.2±3
Xylitol	8.2±1	250±62
D-arabinose	8.1±1	100±10
D-ribose	10.5±2	83±7
L-rhamnose	14±3	33±13
D-galactose	5.3±1	40±5
Sucrose	7.3±1	19±5

### Apo-*Dh*XR Recognizes Carbonyl Substrates Promiscuously in a Cofactor Independent Manner

**Figure 1:**
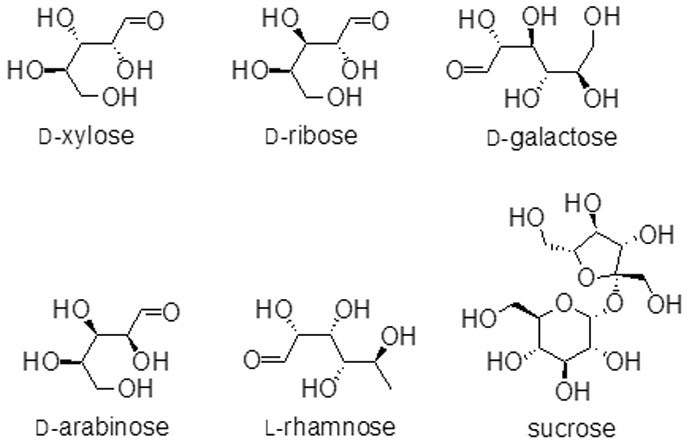
Cartoon representations of carbonyl substrates used in this study.

Xylose reductases have been shown to act on carbonyl substrates but with increased specificity towards D-xylose [45]. We tested the possibility that apo-*Dh*XR may bind to different sugars and isomers of ligands were chosen based on their natural abundance (Fig. 1). Binding of D-ribose, L-rhamnose, D-galactose, D-arabinose, and sucrose to apoenzyme were examined (Fig. S3). D-xylose, D-ribose, D-arabinose are pentose sugars whereas D-galactose and L-rhamnose are hexoses and sucrose is a disaccharide. D-xylose, D-ribose, and D-galactose have the preferred hydroxyl group present at the C2(R) position and are expected to be catalyzed by holo-*Dh*XR [46]. Hydroxyl group at C2(R) position is assumed to favor the transition state binding and therefore essential for catalysis [45]. Our results suggest that all sugars examined in this study show almost similar affinity for the binding of the first sugar molecule to the *Dh*XR. Interestingly, the affinity of D-galactose (K_d_∼5.3 mM) is very similar to that of D-xylose, but D-ribose, a pentose sugar with preferred OH group at the C2(R) position binds with ∼2 fold less affinity (K_d_∼10.5 mM). D-arabinose, a pentose sugar which lacks the expected OH at the C2(R) position but shows higher affinity (K_d,1_∼8.1 mM) and L-rhamnose, a deoxy-hexose sugar binds with equal affinity (K_d,1_∼8.1 mM). Our results indicate that apo-*Dh*XR binds sugars promiscuously and presence of C2(R) hydroxyl group does not contribute to the specificity of ligands binding to the apoenzyme.

### Cofactor Bound Holo-*Dh*XR Selectively Acts on Few Carbonyl Substrates

**Figure 2:**
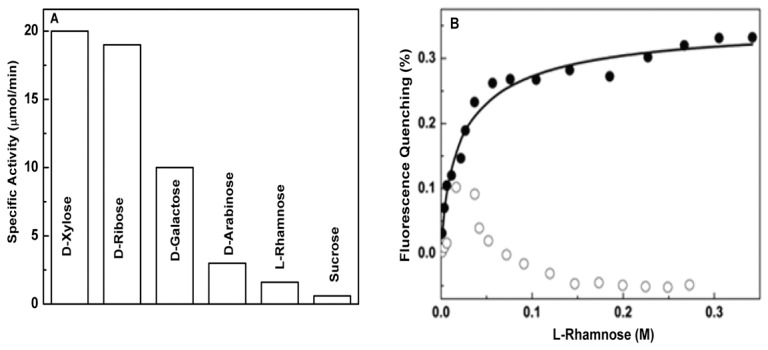
Substrate specificities of *Dh*XR. A) Specific activities of *Dh*XR towards different carbonyl substrates; B) Comparison of L-rhamnose binding to cofactor bound holo-*Dh*XR (○) and apo-*Dh*XR (•); Protein and NADPH concentrations were 2.8×10^−7^ M and 2×10^−5^ M respectively; The pre-formed *Dh*XR-NADPH binary complex was titrated with respective L-rhamnose. Similar amount of enzyme is used for all activity studies.

The indiscriminate sugar binding property of apo-*Dh*XR is not consistent with earlier reports where holo-XR has been shown to preferably reduce substrates with hydroxyl group at C2(R) position [45]. To test whether NADPH bound holoenzyme can reduce substrates promiscuously, we used saturated levels of sugar concentrations to study activity profiles. Our results indicate that holoenzyme shows higher specific activity towards D-xylose as expected, and also shows significant amount of activity towards D-ribose (Fig. 2A). However, it shows significantly less activity for D-galactose and very less or no activity towards other sugars. Although D-galactose, sucrose, and L-rhamnose could bind apo-*Dh*XR with affinity similar to that of D-xylose, holoenzyme activities towards these sugars were reduced significantly (50% for galactose and more than 95% for sucrose and L-rhamnose). These results suggest that holoenzyme, not the apoenzyme has the ability to selectively recognize few substrates.

### Cofactor Bound *Dh*XR-NADPH Complex Selectively Bind and Catalyze D-xylose

To understand the lack of consensus between the binding affinity and catalytic activity, we examined the specific activity of enzyme towards D-xylose in the presence of varied amounts of non-substrate sugars (D-arabinose, L-rhamnose, D-ribose, and D-galactose) which have comparable affinity. Assays were performed at 100 mM D-xylose and concentrations of competing sugars were varied (25 mM to 400 mM). The specific activity towards D-xylose is not inhibited over a range of competing sugar concentrations (Table 2) indicating that both D-arabinose and L-rhamnose cannot compete with D-xylose for the active site of holoenzyme. This observation is in contrast to binding results where L-rhamnose, D-xylose, and D-arabinose can bind the apoenzyme with similar affinity. In the case of D-ribose and D-galactose, the specific activity has increased at higher concentrations, because both of these are catalyzed by *Dh*XR to some extent (table 2). The addition of excess of these compounds increases the concentration of catalyzable substrates. Since holo-*Dh*XR showed little or no activity towards L-rhamnose, we examined the binding of L-rhamnose to holo-*Dh*XR. Both *Dh*XR and NADPH (20.0 µM) are pre-incubated and fluorescence signal was monitored until no further change. The fluorescence signal did not change as we increased the L-rhamnose concentration indicating that L-rhamnose cannot bind to *Dh*XR-NADPH complex (Fig. 2B). In contrast, binding of D-xylose to *Dh*XR-NADPH complex showed further quenching of fluorescence as a function of D-xylose concentration (data not shown). It should be noted that D-xylose is the substrate and is very likely to be reduced soon after binding. However, D-xylose concentration dependent fluorescence quenching of *Dh*XR-NADPH complex confirms that absence of any quenching observed when L-rhamnose is used as ligand is due to the specificity of *Dh*XR-NADPH complex to recognize only a subset of sugars. These results indicate that substrate recognition determinants of holo-*Dh*XR are different and the holoenzyme is more selective in recognizing carbonyl substrates.

**Table 2:** Specific activities of *Dh*XR with xylose in presence of different carbonyl substrates.

	Specific activity (nmol min^−1^)			
Substrate (mM)*	D-arabinose	L-rhamnose	D-ribose	D-galactose
0	72.5±2.4	72.5±2.4	72.5±2.4	72.5±2.4
25	69.4±3.1	76.2±6.4	77.5±3.6	76.8±3.8
100	68.2±3.7	73.4±2.8	84.7±3.8	84.4±3.3
400	66.5±4.5	68.7±3.4	78.5±3.1	99.3±4.3

* amount of substrate added in 100 mM of D-xylose reaction.

### Examination of Apoenzyme-L-rhamnose Complex by Structural Approach

**Figure 3:**
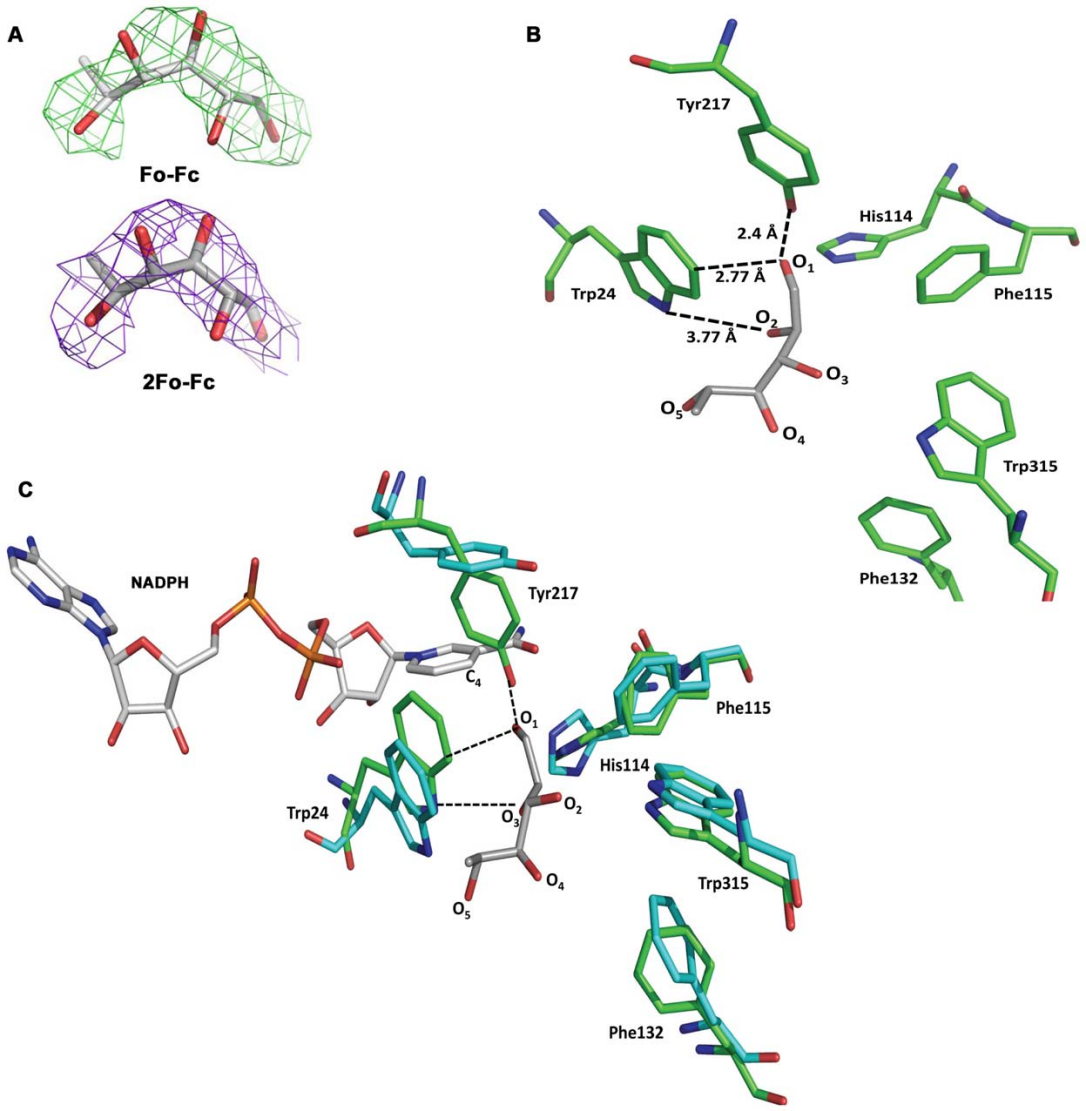
Structural analyses of L-rhamnose interaction with apoenzyme. A) F_o_−F_c_ omit-electron density map (2.5 σ level) shows L-rhamnose backbones and hydroxyl groups and 2 F_o_−F_c_ electron density map (1.0 σ level) also shows rhamnose is bound to active site cavity. B) Interactions of bound L-rhamnose with side chains of residues lining the active site cavity. The aldehyde part of ligand is aligned towards side chain of Y217 with O1 forming strong hydrogen bond with OH of Y217 and also interacting with near by aromatic side chains. C) Superposition of NADPH bound structure to apoenzyme-rhamnose complex. The plane of side chain of Y217 tilted nearly perpendicularly in cofactor bound structure, causing to be atop of NADPH. Apoenzyme-rhamnose complex is shown in green and NADPH bound complex is shown in blue.

To confirm the binding of non-substrate sugars like L-rhamnose to the active site of the apoenzyme, we determined the structure of *Dh*XR in complex with L-rhamnose. Crystal structure of apoenzyme-rhamnose was determined at 3.6 Å and the crystal belongs to space group C2221 with lattice dimensions, a = 135.306, b = 135.281, c = 225.663, α = β = γ = 90°. The final model of *Dh*XR comprises of four monomers as dimers of dimer in an asymmetric unit and two dimers are related by non-crystallographic 2-fold model. The model was refined to reasonable R factors R_work_∼0.26 and R_free_∼0.32 and the stereochemistry checked by PROCHECK indicate 96.0% residues fall into allowed regions. Inspection of initial density maps showed that L-rhamnose was found to bind to one monomer of the dimer. F_o_−F_c_ omit map and 2 F_o_–F_c_ map calculated after initial refinement shows the evidence of L-rhamnose bound to the active site cavity (Fig. 3A). Although X-ray structures are available for apoenzymes and NADPH bound forms, no structure is available for enzyme-substrate/enzyme-ligand complexes.

Analysis of enzyme-L-rhamnose complex reveals that O1 atom of the L-rhamnose forms several hydrogen bonds with residues lining the active site cavity (Fig. 3B). It makes strong hydrogen bonds with OH of Y217 side chain and side chain atoms of W24 and H114, helping to fix the L-rhamnose within the active site pocket. Similarly, O2 of L-rhamnose also interacts with side chain atoms of W24, H114, and D52. Superposition of *Dh*XR-L-rhamnose complex structure onto NADPH bound form of *Ct*XR confirms that L-rhamnose is bound to active site as evidenced from the proximity of L-rhamnose to the nicotinamide ring (Fig. 3C). In addition, the residues predicted to be interacting with D-xylose, W24, H114, and D52 also interact with O2 atom of rhamnose suggesting that D-xylose may also bind to this site though in a different pose. Several notable structural differences were observed in the active site of *Dh*XR-L-rhamnose complex as compared to features of the cofactor bound enzyme. The side chain of Y217 which was found to be stacked with nicotinamide ring of NADPH in the XR-NADPH complex is pushed further into the substrate binding cavity forming the base for L-rhamnose binding. The rotation of the side chain of Y217 by almost 90° to the current position allows O1 of L-rhamnose to make hydrogen bonds with the side chain of Y217, fixing its position in the binding pocket (Fig. 3C). Side chains of aromatic residues W24, F132, W315, and H114 are either rotated or tilted in the rhamnose-apoenzyme structure (Fig. 3C). The arrangement of residues on both sides of bound L-rhamnose shows that the substrate entry channel is lined by hydrophobic residues with bulky side chains, W24 on one side and W315, F132 on the opposite sides. In summary, rhamnose binds to the active site cavity of apoenzyme and structural properties of residues that interact with rhamnose are disturbed upon NADPH binding.

### Specificity of Holo-*Dh*XR is Achieved with Loss of Affinity for Non-substrate Sugars

**Figure 4:**
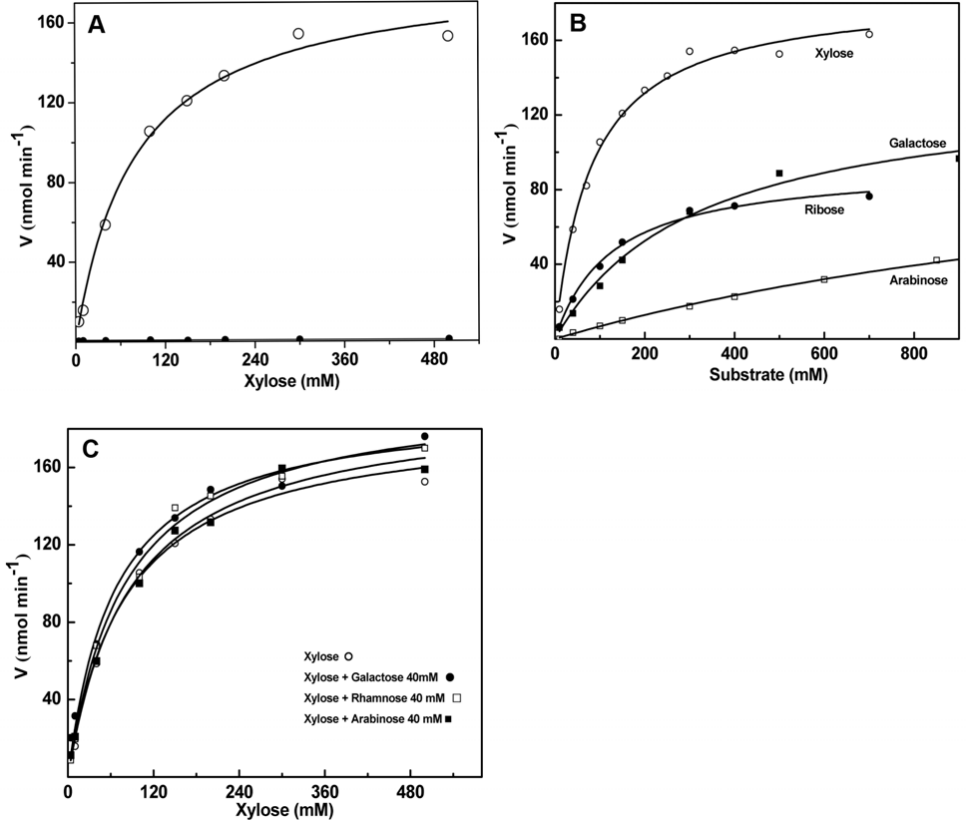
Steady-state kinetic characterization of *Dh*XR. Substrate specificity of *Dh*XR checked and kinetic data were fit to Michaelis-Menten model as described in methods; enzyme concentration is same for all experiments (0.18 µM). A) Kinetic study using D-xylose as substrate; B) Comparative kinetic study using different carbonyl substrates; C) Examination of *Dh*XR kinetic properties towards D-xylose in the presence of fixed amounts (40 mM) of non-xylose substrates.

To understand the specificity determining component in catalysis, we studied the steady state kinetics of holo-*Dh*XR towards different carbonyl substrates. First, we studied the kinetics of D-xylose reduction and fit data to both Hill and Michaelis-Menten models suggesting that magnitude of cooperativity exhibited by dimeric holoenzyme is not significant. Therefore, all kinetic data analyzed fitting to Michaelis-Menten model by Non-Linear Least Squares method. (Fig. 4A). Steady state kinetic parameters are also determined for other sugars (table 3, Fig. 4B). Comparison of kinetic curves of different sugars reveals that D-arabinose was very weakly hydrolyzed and parameters for L-rhamnose could not be estimated due to very low activity. Kinetic parameters indicate that catalytic turnover is not changed much for different substrates, but V_max_ is slightly reduced for non-xylose substrates. Interestingly, K_m_ values are significantly different and the K_m_ of D-arabinose is 22 fold higher than that of D-xylose (table 3). We compared the K_m_/K_d_ ratio as an indicator of net affinity loss for a given substrate (Fig. S5). The net affinity loss for D-arabinose is ∼220 fold compared with 12–15 fold loss for xylose and ribose. Since kinetic parameters for L-rhamnose cannot be estimated due to insignificant catalysis, K_m_/K_d_ ratio could not be estimated, but a minimum of >500 fold is predicted. These results suggest that cofactor binding decreases the affinity for all substrates in general, but relative loss in the affinity is much higher for non-substrate sugars like D-arabinose and L-rhamnose.

**Table 3:** Steady state kinetic parameters for different carbonyl substrates catalyzed by *Dh*XR and kinetic analyses of D-xylose reduction by *Dh*XR in the presence of non-xylose sugars.

Substrate	K_m_ (mM)	V_max_ (nmol min^−1^)	*k*_cat_ (sec^−1^)	*k*_cat_/K_m_ (M^−1^ sec^−1^)
D-xylose	81±6	185.2±4.1	20.6	253.5
D-galactose	320±51	136.5±9.4	15.2	47.4
D-ribose	126±13	93.1±3.2	10.3	82
D-arabinose	1781±135	127.2±6.1	14.1	7.9
D-Xylose+G40mM	66±9	193.3±7.8	21.5	327.9
D-Xylose+A40mM	66±17	178.3±12.1	19.8	302
D-Xylose+R40mM	79±9	199.3±7	22.1	278.5

Note: G, Galactose; A, Arabinose; R, Rhamnose.

In order to further verify that holoenzyme discriminates between substrate and non-substrate sugars, we challenged the catalysis of D-xylose by adding non-substrate sugars during kinetic experiments. The dissociation constants determined for all sugars examined in this study are in the range of 5–8 mM. If *Dh*XR still retains the ability to bind “non-substrate” sugars with similar affinity, kinetic parameters of holoenzyme for D-xylose reduction would be drastically altered in the presence of competing concentrations (≥5 K_d_) of non-xylose sugars. We studied the kinetics of D-xylose reduction by holo-*Dh*XR in the presence of fixed concentration (∼40 mM) of three sugars (Fig. 4C). Analyses of kinetic data show that kinetic parameters essentially remain unaltered when L-rhamnose was used as competing substrate (table 3). Since L-rhamnose and D-arabinose could not be recognized by holoenzyme, K_m_ value remains constant within error limits. Kinetic studies strengthen the results of equilibrium binding and activity studies. Thus, regulating substrate specificity may be an additional role of cofactors in enzyme catalysis.

### Active Site Residues are Dispensable for Ligand Binding Properties of apo-*Dh*XR

**Figure 5:**
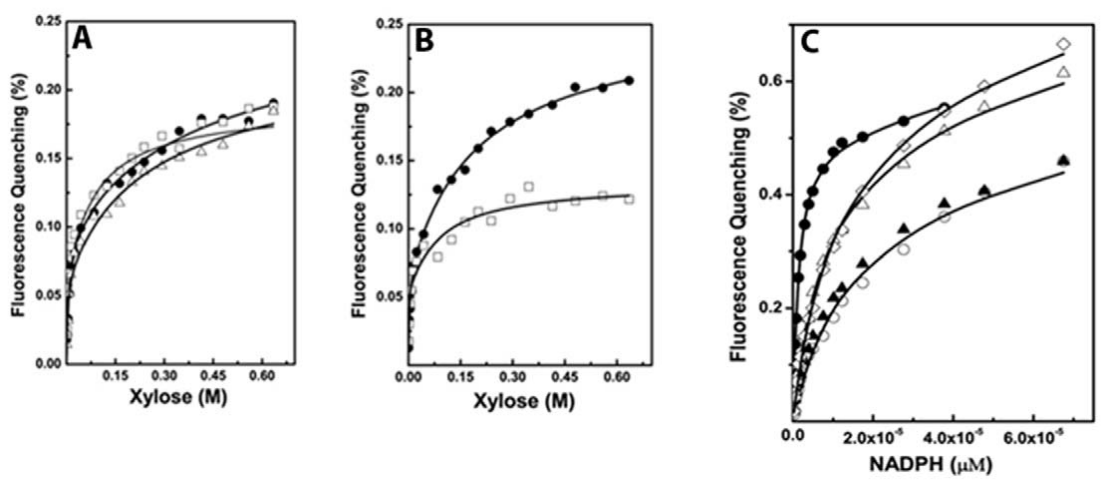
Binding studies of apo-*Dh*XR mutants to various ligands. Protein concentration was 2.8×10^−7^ M for all titration; the data from both titrations were fit to two non-identical site model (eq 1) and results tabulated. A) Fluorescence quenching titrations of *Dh*XR mutants with D-xylose D42A (□); H109A (•); N305A (▵); B) Titrations of mutants with D-xylose; K76A (□); Y47A (•); C) Binding of NADPH to active site mutants; (▴)-Y47A; (•)-H109A; (▵)-D42A; (○)-N305A; (◊)-K76A.

**Figure 6:**
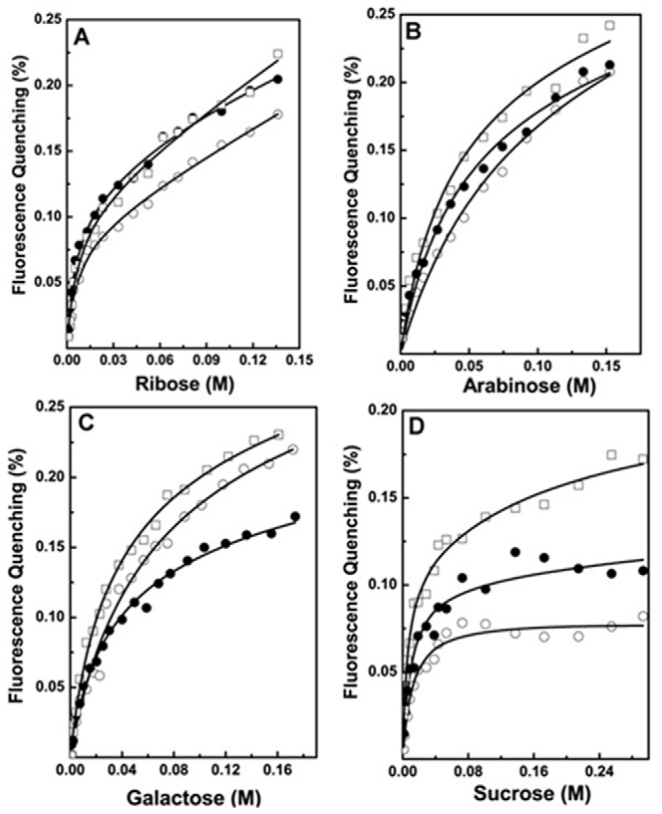
Fluorescence quenching titrations of *Dh*XR mutants with different substrates. H109A (□); K76A (•); Y47A (○); Protein concentration was 2.8×10^−7^ M; the data from both titrations were fit to two non-identical site model (eq 1); A) ribose; B) D-arabinose; C) galactose; D) Sucrose; The solid line represents the best fit to the data.

The highly promiscuous nature of apoenzyme may result from the extended nature of the binding site and many favorable interactions lining the substrate binding pocket [46], [47]. Earlier studies suggested that residues located in the vicinity of substrate and cofactor binding pocket are important for catalysis [32], [46], [47] but effect of mutations on ligand binding by apoenzyme and substrate selectivity are not studied (Fig. S6). We measured the binding affinities of active site mutants for the substrate and cofactor (Fig. 5A–C). CD spectroscopy study showed that purified mutants exhibit secondary structural properties similar to that of wild type protein (data not shown). Interestingly, all mutants can bind D-xylose, although some bind with slightly reduced affinity (table 4). Affinities of D42A and N305A mutants for binding first xylose molecule are similar to that of wild type, but Y47A, K76A, and N305A show reduced affinity. We tested the activity and kinetics of these mutants towards D-xylose and found that all mutants show negligible or no activity (data not shown). Similarly, all mutants can bind NADPH, but showed on average 8–10 fold loss in the affinity for first NADPH molecule binding (Fig. 5C, table 4). Next, we examined the binding affinities of other sugars for active site mutants. Binding isotherms of Y47A, H109A, and K76A mutants binding to ribose, D-arabinose, galactose, and sucrose are shown (Fig. 6A–D, table 5). Y47A mutation reduced the enzyme affinity for D-ribose and sucrose for the first site to ∼ 2 fold, but affinities for galactose and D-arabinose were reduced to 8–10 fold. K76A mutant showed 4–10 fold reduction in the affinity for all sugars except ribose for which the affinity was reduced only 2 fold. Interestingly, H109A showed no reduction in the affinity for sucrose whereas all other mutations resulted in the loss of affinity for sucrose binding. But H109A binds other carbonyl substrates with 2–5 fold reduced affinity suggesting that sucrose, a disaccharide molecule, may bind in a different binding mode and H109 may specifically recognize monosaccharides. We tested the activity of all mutants and activities were normalized to the activity of wild type *Dh*XR with respect to its D-xylose reducing activity. All active site mutants show either no activity or significantly reduced activity towards sugars examined in the study (data not shown). Although active site mutants are competent to bind a variety of sugars as apoenzyme, but lost their activity upon cofactor binding as holoenzyme. In summary, point mutations of active site residues do not abolish ligand binding by apoenzyme, but abolish catalytic activity of the holoenzyme.

**Table 4:** Determination of equilibrium binding constants for D-Xylose and NADPH binding to wild type and active site mutants.

	D-xylose		NADPH	
Strain	Kd_1,int_ (mM)	Kd_2,int_ (mM)	Kd_1,int_ (µM)	Kd_2,int_(µM)
DhXR (WT)	5.3±2	17.2±3	1.1±0.2	3.9±0.2
D10A	6.7±1	333±100	3.3±0.2	10±3
D42A	5.1±0.6	47.6±18	10±0.5	10±0.5
Y47A	12.9±2	333±22	10±0.7	192±26
K76A	12.8±2	500±25	10±0.6	10±0.6
H109	13.3±2	200±80	5.5±2	83±35
N305A	6.4±2	143±40	8.3±2.0	90±25

**Table 5:** Determination of equilibrium binding constants for ligands binding to active site mutants.

	Y47A		K76A		H109A	
Sugars	Kd_1,int_ *	Kd_2,int_ *	Kd_1,int_ *	Kd_2,int_ *	Kd_1,int_ *	Kd_2,int_ *
D-ribose	12±1	166±28	10±2	200±80	13±3	556±30
D-arabinose	66±13	200±40	34±4	290±43	28±3	250±4
D-galactose	40±13	227±100	21±3	250±19	22±2	238±17
Sucrose	11±2	500±75	10±1	500±125	5±0.2	200±120

* all units are in mM.

## Discussion

All structures of AKR family members including the structure of *Dh*XR reported in this study share triosphosphate isomerase (β/α)_8_-barrel (TIM barrel) [23], [29], [46], [48]. Two most important features of enzyme-NADPH complexes structures are; the cofactor invariably binds in a extended conformation and second, it binds via an induced-fit mechanism [29], [46]. Induced-fit binding mechanism is considered to be the basis for changing the affinity of substrate/effector molecules [49]. Binding of nucleotide cofactors have been known to switch proteins from low affinity to high affinity conformations and in some cases switch on and off the protein activity [50], [51]. Although XR catalysis has been studied well and mechanism has been elucidated, biochemical effect of structural rearrangements of the active site has not been studied. In this study, we explored the link between cofactor induced conformational changes within the active site and substrate specificity using xylose reductase as a model system.

Although AKR family enzymes recognize a variety of carbonyl substrates, molecular bases for the differences in substrate recognition among family members are not known [46]. In the absence of structural information on the substrate-enzyme complexes, a systematic study for comparing substrate recognition properties of apo- and holo- enzymes may be useful for understanding the role of cofactor in substrate recognition. As a first step in characterizing the role of cofactors in substrate recognition, we carried out a systematic study to provide evidences for connecting cofactor binding and substrate selectivity. *Dh*XR is a homodimeric enzyme as evidenced from our analytical and structural studies and it reduces D-xylose preferably in a NADPH dependent manner. We provide ample evidence for promiscuous binding of apoenzyme to substrates as well as non-substrates. To confirm the binding of non-substrate molecules to the active site cavity, structure of apoenzyme in complex with L-rhamnose was resolved. L-rhamnose was bound to the active site cavity and binding site was mapped adjacent to the NADPH binding site [29]. In addition, restructuring of active site can be evidenced by comparing rhamnose bound structure with structure of enzyme-NADPH complex. Results of our study clearly indicate that the active site of apoenzyme is more promiscuous and dynamic so as to recognize both cognate substrates and other non-substrate molecules like L-rhamnose. But the active site of cofactor bound holo-*Dh*XR does not recognize non-substrate as evidenced by kinetic studies and competitive inhibition. Although the role of cofactor in catalysis is well known, its role in substrate recognition was not predicted previously.

**Figure 7:**
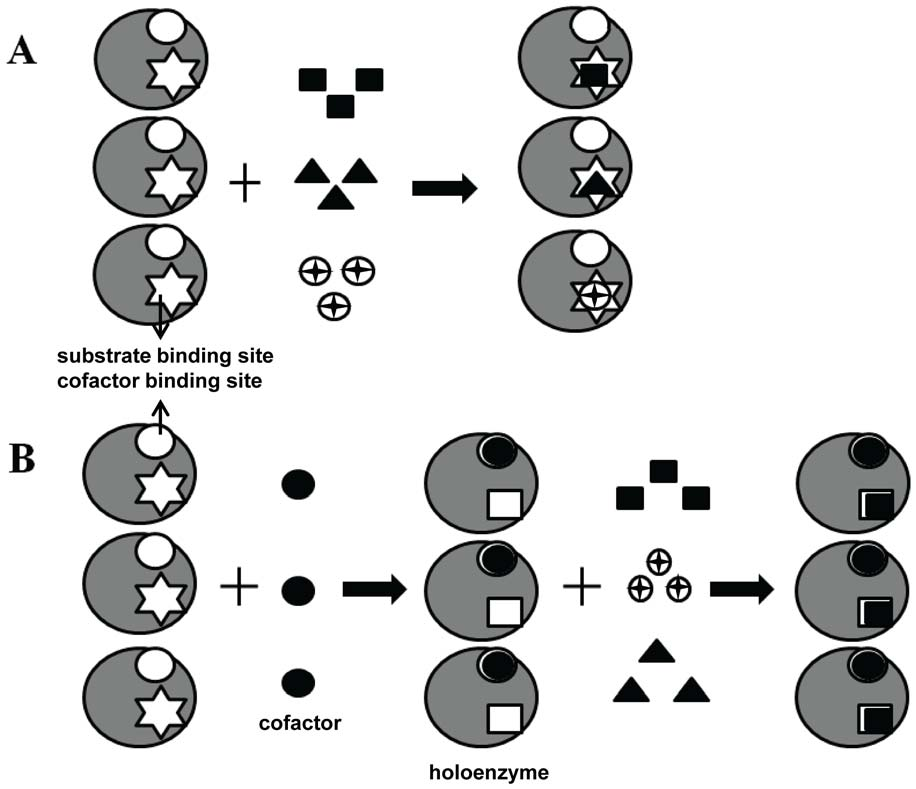
Model for the substrate recognition by *Dh*XR in the absence and in the presence of cofactor NADPH. A) The apo-enzyme recognizes a variety of carbonyl substrates through its extended promiscuous binding site. The cofactor binding site (open circle) and substrate binding site (white star) are shown. Both cofactor and substrates are labeled. B) The binding of cofactor remodels the active site structure to bind a sub set of substrates (D-xylose) selectively.

Pentose and hexose sugars with different stereochemical configurations bind apo-enzyme with very similar affinity. This suggests the preferred hydroxyl group at C2(R) position has either little or no role in apoenzyme-sugar interaction. However, crucial role of the hydroxyl group at C2(R) position in catalysis is confirmed by our activity and steady state kinetic studies, consistent with earlier observations [45]. Non-substrate sugars which showed similar affinity as compared to D-xylose for binding to apoenzyme do not inhibit D-xylose reduction. Our structural studies provide the first glimpse of XR-rhamnose and reveal specificity features of L-rhamnose binding. The ability of apoenzyme to bind L-rhamnose not only stems from hydrogen bonding with OH of Y217 side chain, but also from the disposition of hydrogen bonding and van der Waals interactions between L-rhamnose and non-polar residues with bulky side chains lining the active site cavity (Fig. 3C). Presence of a number of van der Waals contacts between aromatic side chains and L-rhamnose is a typical signature of protein-carbohydrate interactions which is also the basis for accommodating multiple ligands within the active site pocket. The promiscuity of apoenzyme can also be explained by the lack of any interactions between hydroxyl groups of L-rhamnose and main-chain atoms in the active site.

The remodeling of orientations of these aromatic side chains upon NADPH binding as shown in Fig. 3C provides a screening mechanism. Structural characterization of more enzyme-sugar complexes will provide additional insights into different modes of ligand binding and thus aid in development of inhibitors or engineer enzymes with improved selectivity for substrates. Our findings can be interpreted in the context of the structural design of cofactor binding pocket and possible role of cofactor mediated structural dynamics *in vivo*. The high affinity of the cofactor (∼4700 times of substrate affinity) for the apoenzyme may be understood from the structural point of view. Though structure of *Dh*XR is not available for comparison, the induced fit mode of binding of cofactor has been recognized as common feature among AKR family members. Analysis of the structure of C*t*XR reveals that the bound cofactor is locked into the pocket and a number of hydrophobic and polar interactions between nicotinamide ring and residues at the active site stabilize enzyme-NADPH complex [29], [46]. Such high affinity binding of cofactor offers thermodynamic advantage to the enzyme by eliminating the premature formation of any unproductive enzyme-sugar complex. However, the likelihood of unproductive enzyme-sugar complex formation increases if net fluxes of these mono- and disaccharides exceed cofactor flux under *in vivo* conditions. This possibility is demonstrated here by our structural and equilibrium studies which capture the promiscuous binding of apoenzyme. The enzyme utilizes cofactor binding to restrict the flux of non-xylose compounds into the active site and thus, achieves specificity. We provide an array of experimental evidences which point out that cofactor binding has transformed the active site of *Dh*XR to recognize its substrates selectively and changed its ligand recognition properties. This is illustrated in our model where it is shown that NADPH binding allows the *Dh*XR to recognize D-xylose with more specificity (Fig. 7A, B).

Our results presented here suggest that cofactor mediated active site conformational changes may have additional advantages other than preparing the active site geometry for catalysis. Results of activity assays and kinetic studies indicate that activity of *Dh*XR towards D-xylose is not compromised in the presence of non-catalyzable sugars. The interesting feature of our kinetic experiments is that while *k_cat_* and V_max_ for non-xylose substrates remain relatively unchanged, K_m_ for non-substrate sugars increased significantly. The enzyme reaches compromise on losing its affinity for substrate in order to gain specificity. This phenomenon is not reported for any of the AKR family enzymes, although likely to be observed if systematic comparison is made between binding affinities of apoenzyme and holoenzymes. Understanding this cofactor mediated regulatory mechanism is important to understand how AKR family enzymes might achieve their substrate specificity by remodeling their active site cavity. Substrate recognition mechanism of *Dh*XR presents an interesting case where enzyme with simple α_8_/β_8_ fold utilizes its cofactor for both catalysis and substrate screening. The substrate screening mechanism of *Dh*XR is illustrated in Fig. 7A, B. In this model, we propose that the active site of apoenzyme is more dynamic, promiscuous, and it can bind to a variety of carbonyl substrates. In contrast, the cofactor bound binary enzyme complex exhibits an active site which has more defined geometry and can recognize only a subset of carbonyl substrates. Thus, the selectivity of the enzyme increases as cofactor occupies the active site pocket. Structures of apo-*Dh*XR with different carbonyl substrates would reveal the determinants of multi-layered nature of promiscuity.

One of the strengths of the present study is that results presented support a simple model which suggests that cofactor induced structural changes are similar to induced-fit, but the active site is pre-tuned before the substrate binding as compared to the post-substrate binding associated structural changes envisaged by induced-fit model. Enzymes and substrates have to fit to each other as proposed by lock and key model has been used to describe enzyme-substrate interaction in many cases. However, due to the intrinsic flexible nature of active sites, substrate molecules can shape the active site in favor of forward catalysis as proposed by induced-fit theory. In this study, we provide experimental evidences to propose that NADPH binding provides conformational proof reading ability to enzymes in addition to its primary role as hydronium ion donor during the conversion of xylose to xylitol. Further studies of xylose reductases may reveal molecular features of cofactor mediated proof reading ability.

## Supporting Information

Figure S1 **Size-exclusion profile of** ***Dh*** **XR.** Inset shows calibration of column elution volumes using standards as described in results.
(TIF)

Figure S2 **Scan of fluorescence emission of** ***Dh*** **XR at different concentrations of D-xylose.** Excitation was at 292 nm.
(TIF)

Figure S3 **Fluorescence quenching titrations of** ***Dh*** **XR with different carbonyl substrates.** Titrations of ligands binding to *Dh*XR performed in duplicate and protein concentration was 2.8×10^−7^ M. A) D-xylose; B) Xylitol; C) NADPH; D) D-ribose; E) D-arabinose; F) Representative titrations of *Dh*XR with D-Galactose (○); L-rhamnose (•); Sucrose (▵). Results are tabulated (table 1) and solid line represents the best fit to the data.
(TIF)

Figure S4 **Hill plot for the binding of ligands to** ***Dh*** **XR.** Hill plot obtained for D-xylose (○), D-ribose (⋄), and sucrose (×) are shown. The fractional saturation θ equals |F–F_o_|/ΔF_max_, where, F and F_o_ are fluorescence intensities in the presence and absence of ligand, and ΔF_max_ is final fluorescence change. The Hill coefficient, n_H_ is estimated from the data at mid point of saturation.
(TIF)

Figure S5 **Bar graph shows the plot of ratio of K_m_/K_d_ versus sugars.** K_m_ is obtained from steady-state kinetic studies whereas K_d_ is obtained from equilibrium binding studies for ligands binding to apoenzyme.
(TIF)

Figure S6 **Three dimensional ribbon cartoon of the Xylose reductase from** ***C. tenius*** **(PDBID IZ9A).** Active site mutations are labeled in color and residues shown in sticks.
(TIF)

Text S1 **List of primers used for cloning and mutagenesis studies.**
(DOC)
