# Supplementary figures and images for: Co-Factor Binding Confers Substrate Specificity to Xylose Reductase from Debaryomyces hansenii
Source: PLoS One. 2012 Sep 26;7(9):e45525. doi: 10.1371/journal.pone.0045525 (PMC3458928; doi:10.1371/journal.pone.0045525)

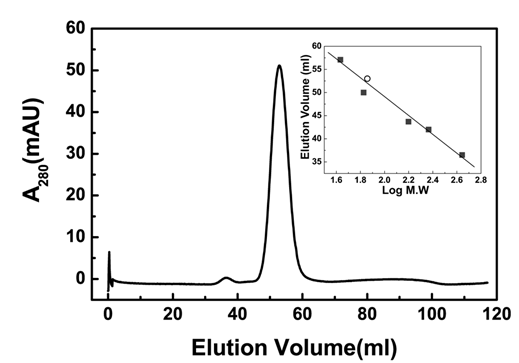

Supplement: Figure S1 — Size-exclusion profile of Dh XR. Inset shows calibration of column elution volumes using standards as described in results. (TIF) [file pone.0045525.s001.tif]

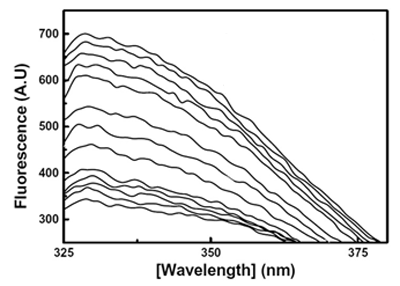

Supplement: Figure S2 — Scan of fluorescence emission of Dh XR at different concentrations of D-xylose. Excitation was at 292 nm. (TIF) [file pone.0045525.s002.tif]

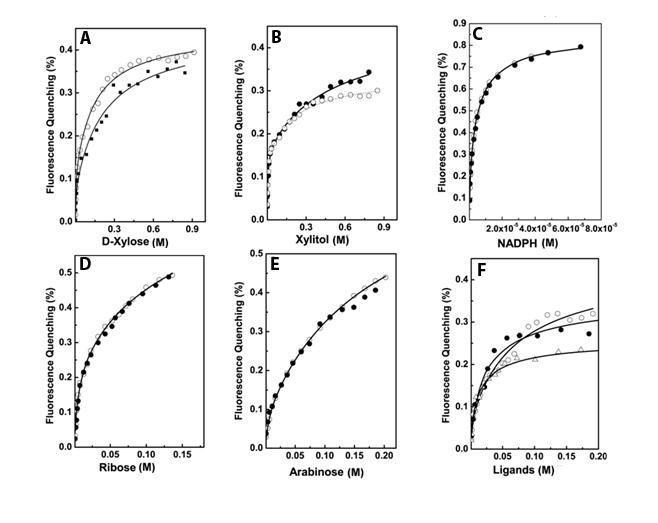

Supplement: Figure S3 — Fluorescence quenching titrations of Dh XR with different carbonyl substrates. Titrations of ligands binding to DhXR performed in duplicate and protein concentration was 2.8×10^−7^ M. A) D-xylose; B) Xylitol; C) NADPH; D) D-ribose; E) D-arabinose; F) Representative titrations of DhXR with D-Galactose (○); L-rhamnose (•); Sucrose (▵). Results are tabulated (table 1) and solid line represents the best fit to the data. (TIF) [file pone.0045525.s003.tif]

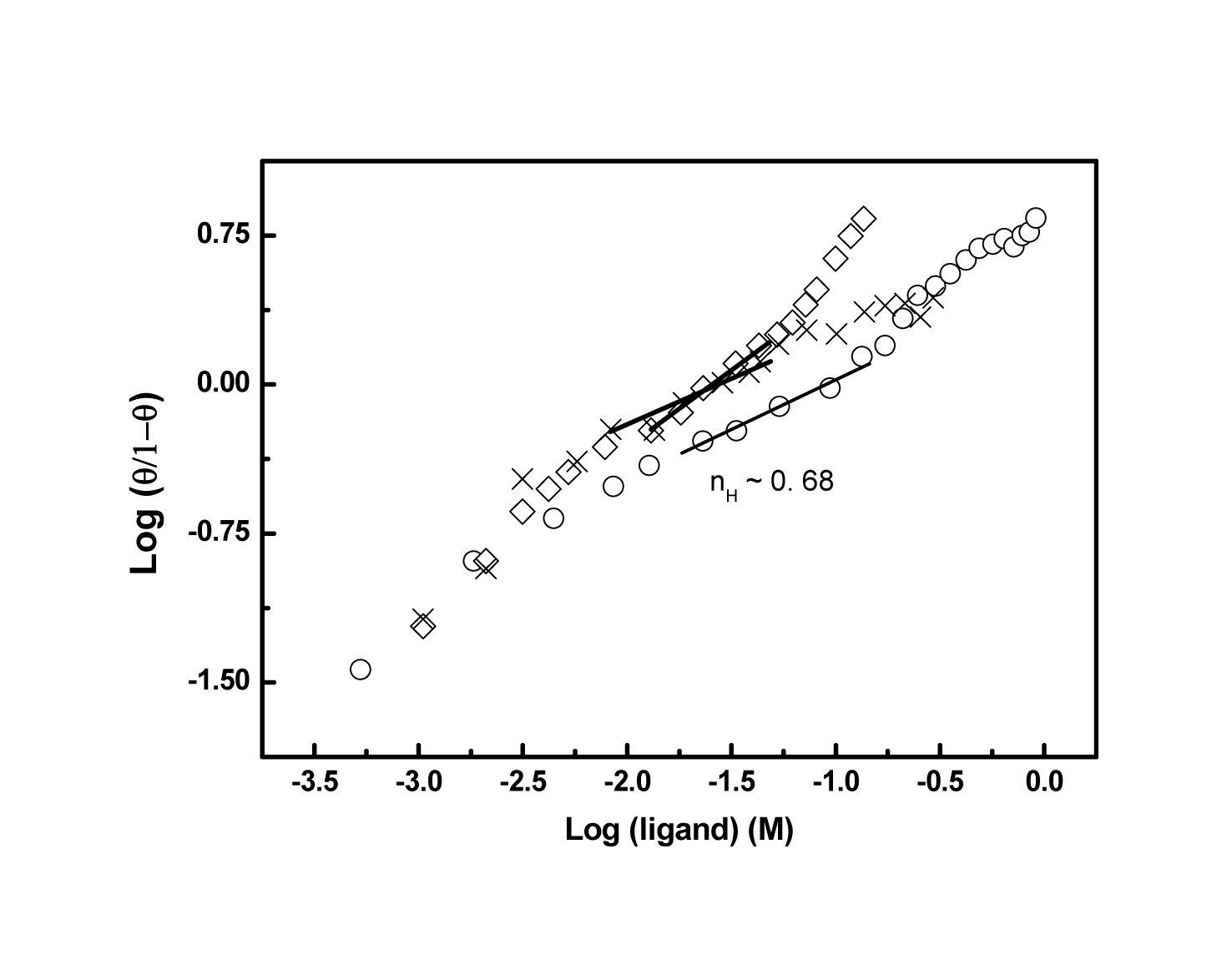

Supplement: Figure S4 — Hill plot for the binding of ligands to Dh XR. Hill plot obtained for D-xylose (○), D-ribose (⋄), and sucrose (×) are shown. The fractional saturation θ equals |F–Fo|/ΔFmax, where, F and Fo are fluorescence intensities in the presence and absence of ligand, and ΔFmax is final fluorescence change. The Hill coefficient, nH is estimated from the data at mid point of saturation. (TIF) [file pone.0045525.s004.tif]

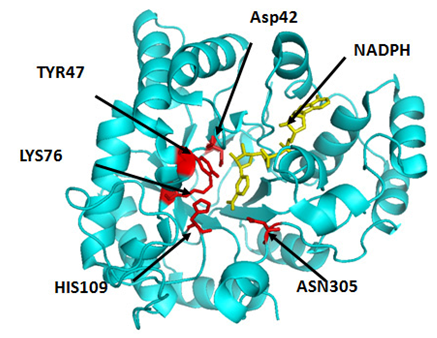

Supplement: Figure S6 — Three dimensional ribbon cartoon of the Xylose reductase from C. tenius (PDBID IZ9A). Active site mutations are labeled in color and residues shown in sticks. (TIF) [file pone.0045525.s006.tif]
