# Supplementary material for: Co-Factor Binding Confers Substrate Specificity to Xylose Reductase from Debaryomyces hansenii
Source: PLoS One. 2012 Sep 26;7(9):e45525. doi: 10.1371/journal.pone.0045525 (PMC3458928; doi:10.1371/journal.pone.0045525)
Supplement: Text S1 — List of primers used for cloning and mutagenesis studies. (DOC) [file pone.0045525.s007.doc]

List of the primers used in the study:

| **Primer Name** | **Primer Sequence 5’-3’** |
| --- | --- |
| pET_XR f | GGAATTCCATATGTCTATTAAGTTAAATTC (with NdeI site) |
| pET XR r | CCGCTCGAGTTAAGCAAAGATTGGAATCT (with XhoI site) |
| D42AF | GTACAGATTATTTGCTGCTGCTCAGGATTACGC |
| D42AR | TAATCCTGAGCAGCAGCAAATAATCTGTACCCAAC |
| Y47AF | CTGCTCAGGATGCCGGTAATTGTAAGGAAATT |
| Y47AR | TTACAATTACCGGCATCCTGAGCAGCATCA |
| K76AF | TGTTCATCACCTCCGCGCTTTGGAACAGTTA |
| K76AR | TAACTGTTCCAAAGCGCGGAGGTGATGAACA |
| H109AF | TTGATCGCTTTCCCAATTGCATTCAAGTTC |
| H109AR | TGGGAAAGCGATCAAGAATAAATCAAGATAGTC |
| N305AF | GAATTAAGATTTGCCAACCCATGGGAT |
| N305AR | ATCCCATGGGTTGGCAAATCTTAATTC |
| W24AF | TTTGGTTGTGCGAAAGTTGAC |
| W24AR | GTCAACTTTCGCACAACCAAA |
